# Circulating Clues in Ménière’s Disease: Elevated Cell-Free DNA and a Pro-Inflammatory Signature in Patients’ Blood

**DOI:** 10.3390/ijms27041948

**Published:** 2026-02-18

**Authors:** Marijana Sekulic, Swethiny Kobivasan, Stavros Giaglis, Daniel Bodmer, Vesna Petkovic

**Affiliations:** 1 Department of Biomedicine, University Hospital Basel, 4031 Basel, Switzerland; 2 Faculty of Medicine, University of Basel, 4056 Basel, Switzerland; 3 Otorhinolaringology Department, University Hospital Basel, 4031 Basel, Switzerland

**Keywords:** Ménière’s disease, cell-free DNA, blood–labyrinth barrier, cytokine, endothelial glycocalyx

## Abstract

Ménière’s disease (MD) is thought to involve dysfunction of the blood–labyrinth barrier, but circulating mechanisms of endothelial injury remain poorly understood. The present study investigated whether cell-free DNA (cfDNA) and inflammatory mediators in plasma contribute to vascular stress and barrier disruption in MD. cfDNA levels were significantly elevated in plasma from patients compared with plasma from healthy controls. Exposure of primary human stria vascularis endothelial cell monolayers to plasma from MD patients led to decreased transepithelial electrical resistance and a significant increase in FITC-dextran permeability, indicating impaired barrier function. MD plasma also induced higher lactate dehydrogenase release and pronounced F-actin disorganization with reduced syndecan-1 expression, consistent with endothelial cytotoxicity and glycocalyx degradation. DNase I partially reversed these effects, implicating extracellular DNA as a key driver. Furthermore, IL-1β, CCL3 (MIP-1α), and CCL27 were elevated in MD plasma. Collectively, our data support a model in which cfDNA and inflammatory mediators cooperatively induce endothelial injury, cytoskeletal remodeling, and glycocalyx shedding, leading to blood–labyrinth barrier weakening. Targeting extracellular DNA or glycocalyx preservation may represent a novel strategy to protect inner ear vascular integrity and modify disease progression in MD, and cfDNA-related readouts may be promising biomarkers of endothelial damage.

## 1. Introduction

Ménière’s disease (MD) is a chronic, relapsing inner ear disorder characterized by episodes of spontaneous vertigo accompanied by fluctuating sensorineural hearing loss, tinnitus, and aural fullness. Although MD was first clinically delineated in the nineteenth century, its etiology and pathophysiology remain incompletely understood [1]. Histopathology studies of human temporal bones provided a crucial early clue, describing marked distension of the endolymphatic spaces, termed endolymphatic hydrops (EH), in patients with typical MD, a finding that was confirmed in larger series [2,3,4]. EH is now considered a histological marker of MD rather than a sufficient causal lesion. The development of delayed gadolinium-enhanced magnetic resonance imaging has enabled in vivo visualization and grading of EH and blood–labyrinth barrier (BLB) permeability [5].

The BLB is a specialized barrier in the stria vascularis comprised of endothelial cells linked by tight junctions, closely associated pericytes (PCs), and perivascular-resident macrophage-like melanocytes. The BLB tightly regulates exchange between the blood and inner ear fluids, maintains the ionic composition of endolymph, and sustains the endocochlear potential essential for normal cochlear and vestibular function [6]. Imaging studies have confirmed that the ears of patients with MD exhibit greater BLB leakage than contralateral or control ears, and that the combined metrics of EH burden and BLB permeability relate to the severity of hearing loss [7,8]. Ultrastructural analyses of the human macula utricle revealed that, compared with normal specimens, MD samples exhibit endothelial swelling, increased pinocytotic vesicles, basement-membrane thickening, and PC alterations in BLB capillaries consistent with chronic barrier injury [9]. Oxidative stress-related proteins and markers of endothelial damage are upregulated in these BLB vessels, further supporting the concept that microvascular dysfunction contributes to inner ear edema and hydrops [10]. In addition, temporal bone studies have reported vascular changes in the stria vascularis of patients with MD [11,12]. Taken together, these data position the stria vascularis and its microvasculature as critical structures at the intersection of fluid homeostasis, perfusion, and tissue injury in MD.

Cell-free DNA (cfDNA) comprises short DNA fragments released into body fluids from apoptotic and necrotic cells, activated immune cells, and, in some settings, extracellular vesicles. Circulating cfDNA has emerged as both a sensitive biomarker of tissue injury and an active driver of sterile inflammation and vascular damage. These DNA fragments include nuclear DNA (nDNA) and mitochondrial DNA (mtDNA), which differ in sequence, structure, and immunostimulatory potential [13]. In a variety of contexts, including cardiovascular, neurological, and critical illness, elevated cfDNA levels correlate with disease severity, organ dysfunction, and mortality, underscoring its relevance as a signal of systemic damage [14].

A major fraction of cfDNA in inflammatory states is derived from neutrophil extracellular traps (NETs), web-like structures composed of DNA, histones, and granular proteins expelled by activated neutrophils [15]. NET-bound and NET-derived cfDNA promote microvascular thrombosis, endothelial activation, and barrier injury in sepsis, thromboinflammatory states, and inflammatory bowel disease [16]. Plasma cfDNA levels track with NET burden and disease severity in these settings and have been proposed to be both a marker and mediator of thromboinflammation [17]. Importantly for the inner ear, NETs have been shown to significantly disrupt barrier integrity in a human BLB model, reducing transepithelial resistance, altering junctional protein organization, and increasing paracellular permeability [18]. These findings suggest that cfDNA/NET-driven vascular damage mechanisms characterized in other organs may be directly relevant to the highly specialized microvasculature of the inner ear.

Recent reviews have highlighted cfDNA among several promising extracellular biomarkers for inner ear diseases, alongside microRNAs, proteins, and metabolites, and discussed lesion-specific changes in cfDNA in experimental models of inner ear injury [19]. Thus, the selective vascular interface between the systemic circulation and perilymph, cfDNA, and NET-associated DNA may not only report on inner ear damage but could also participate in propagating vascular inflammatory injury. However, cfDNA has not yet been systematically investigated in MD, and its potential contributions to vascular dysfunction and inflammatory endotypes in MD remain unexplored.

Beyond purely hydropic and vascular hypotheses, converging clinical, immunological, and genetic evidence now supports the view that, at least in a substantial subset of patients, MD behaves as a chronic immune-mediated inflammatory inner ear disorder [20,21]. Local inflammatory activity has also been documented within inner ear-related tissues. Analysis of endolymphatic sac luminal fluid from patients with MD demonstrated increased expression of interferon (IFN)-γ, interleukin (IL)-6, and tumor necrosis factor (TNF)-α compared with controls, supporting the concept that immune-mediated inflammation of the endolymphatic sac contributes to disease mechanisms [22]. The specific patterns of circulating inflammatory factors associated with MD and their relationship with clinical phenotype, hydrops burden, and BLB integrity remain incompletely defined. Therefore, a more detailed characterization of cytokine and chemokine profiles in MD is essential to refine pathophysiological models, identify biologically meaningful patient subgroups, and explore targeted anti-inflammatory or barrier-stabilizing therapies.

In this study, we aimed to investigate whether circulating factors in the plasma of patients with MD, particularly cfDNA and inflammatory mediators, contribute to endothelial stress and BLB dysfunction by examining their impact on key features of endothelial health, including barrier integrity, cytotoxicity, cytoskeletal organization, and glycocalyx structure.

## 2. Results

### 2.1. Elevated Circulating cfDNA in Ménière’s Disease

**Figure 1:**
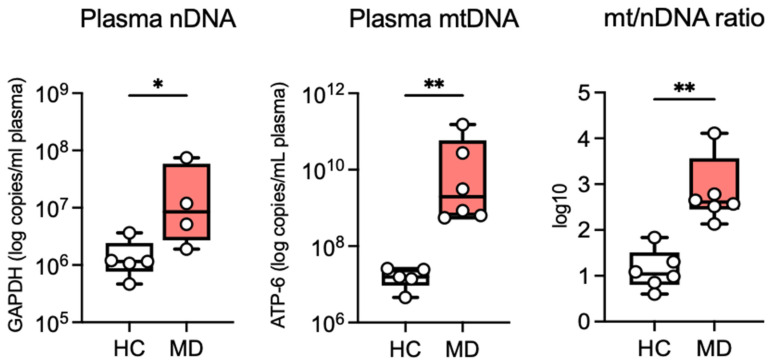
Elevated plasma nuclear DNA (nDNA) and mitochondrial (mtDNA) levels in patients with Ménière’s disease (MD). Quantification of circulating cell-free DNA (cfDNA) and mitochondrial-to-nuclear cfDNA ratio in plasma from healthy controls (HCs) and patients with MD. Each point represents an individual participant. * *p* < 0.05, ** *p* < 0.01. Data are presented as the mean ± SD. Experiments were run in triplicate (*n* = 3).

To investigate whether plasma from patients with MD exhibits signatures of cellular stress or damage, we quantified the cell-free nDNA and mtDNA levels in plasma samples from patients and healthy controls (HCs). The cfDNA concentrations were significantly increased in patients with MD compared with HCs. The mtDNA levels showed an approximately 2-log elevation in MD samples (*p* < 0.01), whereas nDNA levels were moderately but significantly higher (*p* < 0.05; Figure 1). To further explore potential cfDNA release mechanisms, we calculated the mitochondrial-to-nuclear DNA (mtDNA:nDNA) ratio in plasma samples. This ratio provides an index of the relative contribution of mitochondrial versus nuclear DNA to circulating cfDNA. Patients with Ménière’s disease exhibited a significantly altered mtDNA:nDNA ratio compared with controls (*p* < 0.01; Figure 1), suggesting that cfDNA release may not be solely attributable to nonspecific tissue damage, but could involve active, regulated immune processes. These results indicate enhanced abundance of cell-free DNA in the circulation of patients with MD.

### 2.2. Plasma from Ménière’s Disease Impairs Barrier Integrity and Increases Permeability in the BLB Model

**Figure 2:**
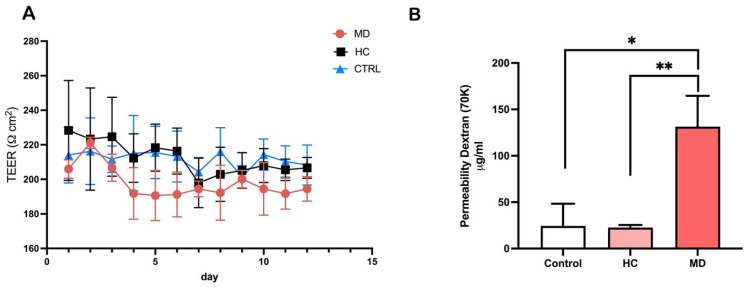
Plasma from patients with Ménière’s disease (MD) disrupts blood–labyrinth barrier integrity and increases permeability. (**A**) Time course of trans-endothelial electrical resistance (TEER) measurements in chips containing primary stria vascularis endothelial cells treated with plasma from patients with MD or healthy controls (HCs) or in untreated controls (CTRL). MD plasma caused a sustained reduction in TEER compared to HCs or the CTRL condition. (**B**) FITC-dextran (70 kDa) permeability assay showing increased paracellular permeability in cells treated with MD plasma. Data are presented as the mean ± SD. * *p* < 0.05, ** *p* < 0.01. Number of individual experiments with all treatment conditions (*n* = 3).

To evaluate the effects of plasma from patients with MD on BLB function, we cultured primary human stria vascularis endothelial cells and PC in a 3D Transwell system and monitored TEER and macromolecular permeability. TEER values were recorded over a 14-day period following exposure to plasma from patients with MD or HCs or to untreated medium (control) (Figure 2A). Compared to HCs and control conditions, cells treated with MD plasma exhibited a consistent trend of reduced TEER. Although it was not significant, this declining trend became apparent within the first few days of exposure and persisted throughout the experimental period.

FITC-dextran (70 kDa) permeability assays were performed to further assess barrier function (Figure 2B). Endothelial cell monolayers exposed to MD plasma demonstrated a significant increase in dextran permeability compared to both HC plasma-treated and control conditions (*p* < 0.05 and *p* < 0.01, respectively). These findings demonstrate that circulating factors in plasma from patients with MD could compromise the endothelial integrity of BLB in the stria vascularis, supporting a link between systemic inflammation and inner ear barrier dysfunction.

### 2.3. DNase I Treatment Partially Attenuates MD Plasma-Induced Cytotoxicity and Preserves Endothelial Morphology

To investigate whether extracellular DNA contributes to the damaging effects of MD plasma on endothelial cells, primary human stria vascularis endothelial cells were treated with 3% plasma from patients with MD or HCs with or without DNase I (30 IU) supplementation. Cytotoxicity was assessed by LDH release, and cytoskeletal organization was evaluated by phalloidin staining (F-actin).

**Figure 3:**
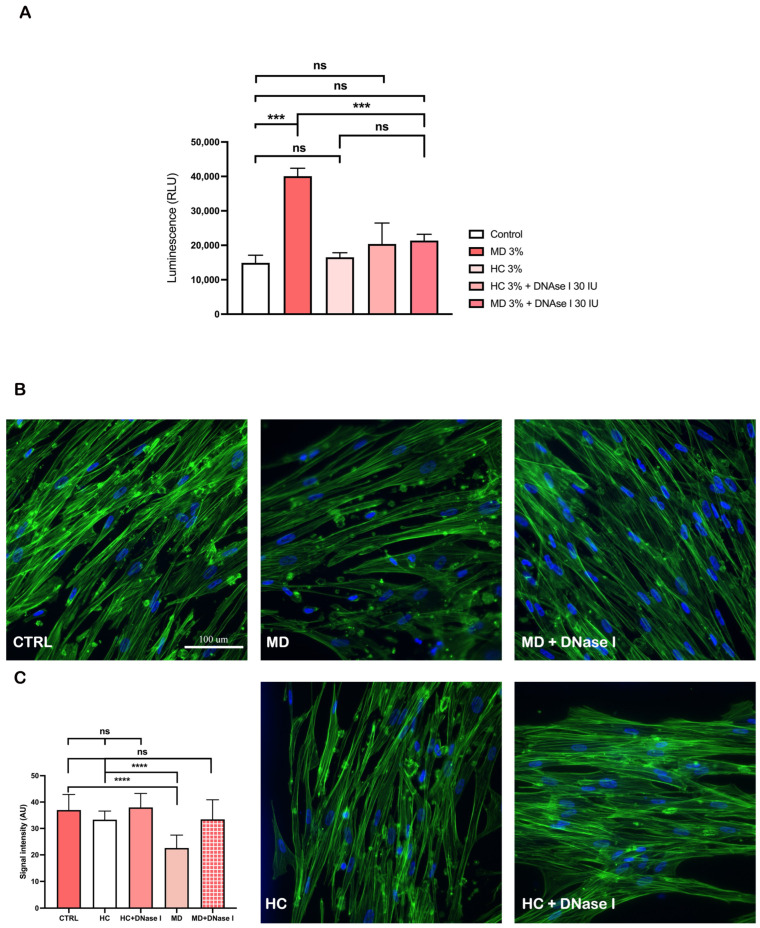
DNase I partially reverses plasma-induced cytotoxicity and cytoskeletal disruption in primary human stria vascularis endothelial cells. (**A**) Lactate dehydrogenase cytotoxicity assay showing increased cell damage following exposure to 3% plasma from patients with Ménière’s disease (MD) compared to untreated controls. Co-treatment with DNase I (30 IU) slightly reduced lactate dehydrogenase release. (**B**) Representative phalloidin (F-actin, green) and DAPI (nuclei, blue) staining showing disorganization of actin filaments in MD plasma-treated cells, with partial restoration of the cytoskeletal structure upon DNase I treatment. Scale bar = 100 μm. (**C**) Quantification of the fluorescence signal intensity confirming significant loss of F-actin organization in MD plasma-treated cells relative to control conditions (CTRL) and healthy controls (HCs). Number of individual experiments with all treatment conditions (*n* = 3). Data are presented as the mean ± SD. *** *p* < 0.001, **** *p* < 0.0001; ns, not significant.

Exposure to MD plasma resulted in a modest but significant increase in LDH release compared with untreated control cultures (Figure 3A), indicating higher levels of cytotoxicity. The addition of DNase I slightly reduced LDH levels in MD plasma-treated cells, suggesting a potential protective role of DNase I against DNA-associated damage. In contrast, treatment with HC plasma did not significantly alter LDH release relative to control conditions, regardless of whether DNase I was present.

Phalloidin staining revealed clear alterations in cell morphology following exposure to MD plasma (Figure 3B). MD plasma-treated endothelial cells exhibited disorganized actin filaments and reduced fluorescence intensity consistent with cytoskeletal disruption. In contrast, DNase I co-treatment partially restored the organized F-actin network and preserved cell alignment. Quantitative analysis of the fluorescence signal intensity (Figure 3C) confirmed a significant reduction in actin organization in MD plasma-treated samples compared to control, HC, and HC + DNase I conditions (*p* < 0.0001). These findings suggest that extracellular DNA present in the plasma of patients with MD contributes to cytoskeletal destabilization and mild cytotoxicity, and DNase I treatment may mitigate these effects.

### 2.4. DNase I Treatment Partially Restores Syndecan-1 Expression in Endothelial Cells

**Figure 4:**
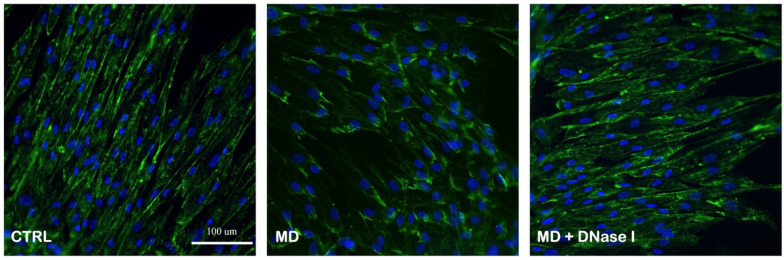

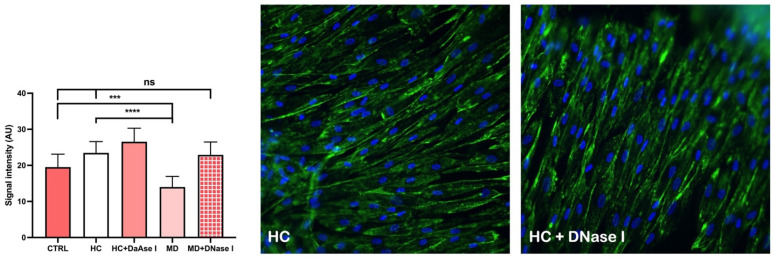
DNase I partially restores syndecan-1 expression in endothelial cells exposed to plasma from patients with Ménière’s disease (MD). Primary human stria vascularis endothelial cells were treated with 3% plasma from patients with MD or healthy controls (HCs), with or without DNase I (30 IU). Cells were stained for syndecan-1 (green) and nuclei (DAPI, blue) to visualize glycocalyx integrity. MD plasma caused a pronounced loss and disorganization of the syndecan-1 signal compared to control (CTRL) and HC plasma-treated cells, whereas DNase I co-treatment partially restored the syndecan-1 distribution. Scale bar = 100 μm. Bottom left: Quantitative analysis confirmed significantly decreased syndecan-1 signal intensity in MD plasma-treated cells. *** *p* < 0.001, **** *p* < 0.0001; ns, not significant. Number of individual experiments with all treatment conditions (*n* = 3). Data are presented as the mean ± SD.

To assess whether plasma from patients with MD affects the integrity of the endothelial glycocalyx, we analyzed syndecan-1 expression in primary human stria vascularis endothelial cells after exposure to 3% plasma from patients with MD or HCs, with or without DNase I (30 IU) treatment. Endothelial cells exposed to MD plasma exhibited a marked reduction in the fluorescence intensity of syndecan-1 and a disrupted membrane localization pattern (Figure 4), indicating degradation of the glycocalyx layer. In contrast, HC plasma maintained strong, continuous syndecan-1 staining along the cell surface, similar to untreated controls. The addition of DNase I to MD plasma resulted in partial recovery of the syndecan-1 signal and improved cell morphology, suggesting that extracellular DNA contributes to the MD plasma-induced glycocalyx damage.

Quantitative analysis of the fluorescence intensity (Figure 4) confirmed that syndecan-1 expression was significantly reduced in MD plasma-treated cells compared to control conditions or the HC plasma and HC plasma + DNase I groups. DNase I co-treatment moderately increased syndecan-1 levels, though the difference did not reach significance compared with MD plasma alone. These findings support the hypothesis that extracellular DNA plays a role in endothelial glycocalyx degradation in MD and that enzymatic degradation of circulating DNA may help preserve barrier integrity.

### 2.5. Cytokine-Chemokine Imprint of MD: Elevated Plasma IL-1β, CCL3, and CCL27

**Figure 5:**
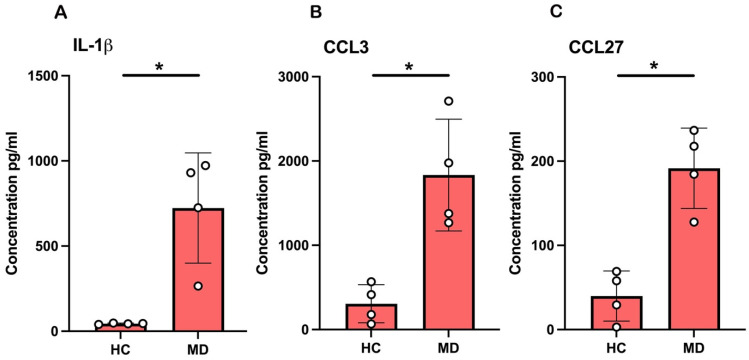
Pro-inflammatory cytokines and chemokines increased in plasma from patients with Ménière’s disease (MD). Plasma concentrations of (**A**) IL-1β, (**B**) CCL3/MIP-1α, and (**C**) CCL27 were measured in healthy controls (HCs) and patients with MD. Data are presented as mean ± SD; *n* = 4. * *p* < 0.05. Individual experiments were performed in triplicate.

Plasma concentrations of IL-1β, CCL3, and CCL27 were consistently higher in patients with MD than in HCs. IL-1β levels were low in plasma from HCs, whereas samples from patients with MD presented a several-fold increase with greater inter-individual variability (Figure 5A). A similar pattern was observed for CCL3, with MD plasma exhibiting elevated chemokine levels compared with HC plasma (Figure 5B). CCL-27 showed the most pronounced difference, with markedly increased concentrations in MD plasma and a minimal signal in HC plasma (Figure 5C). Taken together, the findings indicate that MD is accompanied by a systemic pro-inflammatory cytokine/chemokine profile in the circulation.

## 3. Discussion

To investigate whether plasma from patients with MD exhibits pro-inflammatory signatures of cellular stress or damage, we quantified the cell-free nDNA and mtDNA levels in plasma samples from patients with MD and HCs, as cfDNA is known to be damaging to endothelial cells [23,24]. Increased cell-free DNA (nDNA and mtDNA) concentrations in plasma from patients compared with HCs indicated enhanced release of cfDNA into the circulation in MD. mtDNA is recognized as a damage-associated molecular pattern (DAMP) that can activate innate immune receptors, such as Toll-like receptor 9 (TLR9) and cyclic GMP-AMP synthase (cGAS)-STING pathways, leading to the production of inflammatory cytokines and type I interferons [25,26,27,28]. Elevated cfDNA levels have been detected in several inflammatory and vascular disorders, including sepsis, atherosclerosis, and autoimmune diseases [29], where they contribute to endothelial activation, increased permeability, and glycocalyx shedding [30,31].

Furthermore, our data demonstrated that exposure to plasma from patients with MD leads to measurable functional alterations in primary human stria vascularis endothelial cells, suggesting that circulating factors in the plasma of patients may compromise inner ear barrier integrity. Although the decrease in TEER did not reach significance, a consistent downward trend in MD plasma-treated samples was observed over time, suggesting, but not conclusively demonstrating, early barrier impairment. Accordingly, TEER data should be interpreted as indicative of a trend rather than definitive evidence of barrier breakdown. In contrast, FITC-dextran permeability assays revealed a statistically significant increase in macromolecular leakage across the endothelial monolayer in response to MD plasma, providing functional evidence of disrupted barrier selectivity. These findings point to plasma-induced endothelial dysfunction as a potential contributor to the vascular and fluid dysregulation observed in MD. Reasons for this effect of plasma on the endothelial barrier in our study could be excess DNA release, which is in itself inflammatory, or could be pro-inflammatory cytokines, as previously shown in our and other studies [18,20,32,33].

Disruption of endothelial barrier integrity has been recognized as a hallmark of inflammatory microenvironments in multiple organs, including the blood-brain barrier and blood-retinal barrier [34,35]. In the context of the inner ear, such cfDNA-mediated inflammation could disrupt the BLB, similar to how circulating DAMPs compromise the blood-brain barrier under neuroinflammatory conditions [36]. Our findings of increased circulating nDNA and mtDNA levels in patients with MD support this mechanism and suggest that cfDNA may contribute to endothelial stress and barrier dysfunction through both direct immune activation and secondary cytokine amplification. The mtDNA:nDNA ratio provides additional insight into the potential cellular and mechanistic origins of circulating cfDNA in Ménière’s disease. A relative enrichment of mtDNA is increasingly linked to active immune-mediated DNA release rather than passive apoptotic or necrotic processes. In this context, neutrophil extracellular traps (NETs) represent a particularly relevant source, as NET formation entails the extrusion of both nuclear and mitochondrial DNA with potent immunostimulatory properties. Moreover, recent evidence demonstrates that cooperative mitochondrial DNA release from platelets and neutrophils can drive type I interferon signalling in systemic inflammatory disease, supporting a mechanistic role for immune-cell-derived mtDNA in disease amplification [28]. Although NET-associated markers or platelet-neutrophil interactions were not directly assessed in the present study, the observed cfDNA profile and mtDNA:nDNA ratio are compatible with such active immune pathways. These findings support further investigation of immune-mediated cfDNA release mechanisms in Ménière’s disease.

To further dissect the mechanisms underlying plasma-induced endothelial injury in MD, we examined whether extracellular DNA contributes to endothelial cytotoxicity and cytoskeletal alterations in primary human stria vascularis endothelial cells by introducing DNase I. Exposure to 3% MD plasma induced a significant increase in LDH release, indicative of enhanced cell membrane damage and cytotoxicity, whereas DNase I treatment partially mitigated this effect. In parallel, phalloidin staining of F-actin revealed disrupted cytoskeletal organization in cells treated with MD plasma compared to HC plasma or untreated controls, which was characterized by reduced fiber alignment and actin intensity. Quantitative fluorescence analysis confirmed a significant reduction in the actin signal in the MD group, whereas co-treatment with DNase I partly restored cytoskeletal integrity. These findings suggest that extracellular DNA present in MD plasma contributes to endothelial damage and actin disorganization, potentially via pro-inflammatory and stress-mediated mechanisms. In endothelial systems, cfDNA induces oxidative stress, promotes cytoskeletal retraction, and increases barrier permeability—effects that have been demonstrated in vascular and pulmonary endothelial cells [37]. DNase I, an endonuclease that degrades extracellular DNA, has been shown to reduce inflammation and vascular leakage in sepsis and ischemia models, aligning with the partial normalization of cytoskeletal structure we observed upon DNase I treatment [38,39,40]. The observed cytoskeletal disruption in MD plasma-treated endothelial cells likely reflects glycocalyx degradation and loss of integrity of mechanotransduction, both of which are crucial for maintaining the selective permeability of the BLB.

Our results further demonstrated that plasma from patients with MD leads to a significant reduction in syndecan-1 expression in primary human stria vascularis endothelial cells, indicative of endothelial glycocalyx disruption. Syndecan-1, a major heparan sulfate proteoglycan located on the endothelial surface, is essential for maintaining vascular barrier integrity, mechanotransduction, and anti-inflammatory balance [41,42]. In our model, exposure to MD plasma resulted in decreased syndecan-1 fluorescence intensity and loss of continuous membrane localization, suggesting shedding or degradation of the glycocalyx. Treatment with DNase I partially restored syndecan-1 expression and cell morphology, supporting the hypothesis that extracellular DNA (i.e., cfDNA) contributes to glycocalyx injury. Shedding of syndecan-1 and other glycocalyx components is a well-established marker of endothelial injury in inflammatory and vascular disorders. Elevated circulating syndecan-1 levels have been reported in conditions such as sepsis, diabetes, and ischemia-reperfusion injury, where oxidative stress and pro-inflammatory cytokines, especially TNF-α and IL-1β, activate matrix metalloproteinases (MMPs) and heparanases that cleave syndecan ectodomains [43,44]. Our findings are consistent with this mechanism and suggest that a similar inflammatory glycocalyx degradation process occurs in the BLB of patients with MD.

Importantly, cfDNA itself has been implicated in promoting endothelial glycocalyx breakdown. Studies have shown that mtDNA and NETs can directly damage the glycocalyx through TLR9-dependent activation and release of reactive oxygen species, resulting in actin reorganization and loss of the surface expression of syndecan-1 [45,46]. The partial restoration of syndecan-1 levels after DNase I treatment in our study supports a model in which cfDNA acts as a DAMP, perpetuating endothelial inflammation and glycocalyx injury, which can be alleviated by enzymatic degradation of extracellular DNA.

In plasma from patients with MD, we observed elevated IL-1β together with increased levels of chemokines CCL3 (MIP-1α) and CCL27, indicating a systemic pro-inflammatory environment. IL-1β is a prototypical upstream “master switch” cytokine of innate inflammation and is produced mainly by monocytes/macrophages and other innate immune cells; it is known to orchestrate downstream cytokine cascades and tissue inflammation [47,48]. CCL3 is a strongly pro-inflammatory chemokine that recruits and activates monocytes, T cells, and neutrophils via CCR1/CCR5 and critically mediates leukocyte trafficking in inflamed tissues, including neutrophil recruitment in immune inflammation [49]. Moreover, CCL3 has been implicated in NETosis-linked feedback loops, where IL-1β-driven CCL3 production promotes neutrophil activation and NET formation, further amplifying local inflammation [50,51]. The number of patients with MD included in this study was limited, which may restrict the generalizability of these findings. Despite the small cohort size, all patients presented with a typical MD phenotype, including recurrent, often disabling vertigo attacks, fluctuating low-frequency hearing loss, tinnitus, and aural fullness. All patients had bilateral disease involvement and had previously received standard symptomatic therapies, including antivertigo medication, headache treatment, and corticosteroids. However, at the time of blood sampling, none of the patients were receiving medication. This shared pattern of recurrent vestibulocochlear symptoms, sometimes associated with infections, inflammation, or stress, indicates a relatively homogeneous clinical subgroup, which may have facilitated the detection of cfDNA-induced and plasma-induced endothelial changes in our model. Nevertheless, future studies incorporating larger and clinically diverse MD cohorts will be essential to validate these findings, assess inter-patient variability, and determine their broader relevance to MD pathophysiology.

Collectively, these results provide strong evidence that cfDNA and inflammatory mediators present in the plasma of patients with MD cooperatively induce endothelial glycocalyx disruption, leading to barrier weakening and cytoskeletal disorganization. The partial rescue of these phenotypes by DNase I highlights the potential therapeutic relevance of targeting extracellular DNA-driven inflammation to preserve endothelial and barrier integrity in the inner ear. Future studies should investigate whether cfDNA levels or inflammatory mediators could serve as biomarkers of endothelial injury and disease severity in MD and whether therapeutic strategies targeting extracellular DNA or glycocalyx restoration could mitigate BLB damage and potentially alleviate MD symptoms.

## 4. Materials and Methods

### 4.1. Human Tissue Collection, Cell Isolation, and Culture

Human stria vascularis cells were isolated, maintained, and differentiated from post-mortem human temporal bones obtained from the Institute of Pathology, Basel, Switzerland (ethical approval was obtained from the Ethics Committee of Northwestern and Central Switzerland (EKNZ). Protocol code: 2020-01379; date of approval: 31 August 2023). Autopsy-derived temporal bones were used as the tissue source due to the limited availability of healthy stria vascularis tissue from surgical procedures. Donors (*n* = 6) were 50–75 years of age. The dissection of healthy stria vascularis tissue was performed as described previously [32]. Briefly, freshly isolated tissue sections were immediately transferred into either human endothelial cell or PC medium (ScienCell, Carlsbad, CA, USA, cat# 1001 and cat# 1201, respectively) for transport.

Upon arrival in the laboratory, tissue samples were minced into small fragments and enzymatically dissociated using 0.25% trypsin (Sigma Aldrich, St. Louis, MO, USA, cat# T4049) for 5 min at 37 °C. The reaction was stopped by the addition of soybean trypsin inhibitor (Defined Trypsin Inhibitor, Gibco, Waltham, MA, USA, cat# R007100). Tissue fragments were then vigorously pipetted at least 30 times to further dissociate the cells, and the suspension was centrifuged at 1100 rpm for 10 min. After removal of the supernatant, the cell pellet was resuspended in the appropriate culture medium and seeded into 24-well plates pre-coated with human fibronectin for endothelial cell cultures or poly-L-lysine for PC cultures (Sigma Aldrich, cat# F0895).

### 4.2. Cell Culture and Treatment

Cells were maintained at 37 °C in a humidified atmosphere of 5% CO_2_. The endothelial cell growth medium consisted of 500 mL endothelial cell basal medium, 5 mL endothelial cell growth supplement (ScienCell, cat# 1052), and 5 mL penicillin/streptomycin solution (ScienCell, cat# 0503). The PC growth medium consisted of 500 mL PC basal medium, 5 mL PC growth supplement (ScienCell, cat# 1252), and 5 mL penicillin/streptomycin solution (ScienCell, cat# 0503). Prior to experimental use, cells were expanded in T25 or T75 flasks coated with the appropriate attachment factor (human fibronectin for endothelial cells, poly-L-lysine for PCs). Cells at passage 2 or 3 were used for all experiments. For seeding, subconfluent cultures were detached with trypsin, pelleted, and resuspended in the respective growth medium at the desired cell density.

For transendothelial electrical resistance (TEER) and permeability assays, cells were seeded onto 24-well Transwell^®^ inserts for 3D culture as described in the TEER measurements section below and, after establishment of a confluent monolayer, treated with 3% (*v*/*v*) plasma from healthy controls (HCs) or patients with MD or with vehicle (control) for 72 h (day 3–6 post-seeding).

For lactate dehydrogenase (LDH), phalloidin, and syndecan-1 experiments, endothelial cells were treated for 72 h with 3% (*v*/*v*) plasma from HCs or patients with MD or with the same plasma conditions in combination with DNase I (30 IU; Sigma Aldrich, cat# AMPD1). Control cells received vehicle only.

### 4.3. Plasma Collection and Quantification of Circulating cfDNA

Peripheral venous blood (7 mL) was collected from four patients with MD (ethical approval was obtained from the Ethics Committee of Northwestern and Central Switzerland (EKNZ). Protocol code: 2023-01154; date of approval: 7 August 2023). and four healthy donors (blood bank) in EDTA tubes. Platelet-poor plasma (PPP) was prepared within 4 h by centrifugation at 1200 *g* for 10 min and 16,000 *g* for 10 min at room temperature. The PPP was carefully aspirated without disturbing the buffy coat, aliquoted, and stored at −80 °C.

Total cfDNA was extracted from 500 µL PPP using the QIAamp DNA Blood Mini Kit (Qiagen, Hilden, Germany) and eluted according to the manufacturer’s instructions. DNA concentration and purity were assessed on a NanoDrop ND-1000 spectrophotometer (NanoDrop Technologies, Wilmington, DE, USA), and eluates were stored at −20 °C until further evaluation.

The mtDNA and nDNA copy numbers were assessed as previously described [52]. In brief, copy numbers were quantified by quantitative real-time PCR on an Applied Biosystems StepOnePlus system. The mtDNA ATP-6 region (nt 8981–9061) was amplified using primers 5′-ACCAATAGCCCTGGCCGTAC-3′ and the nDNA GAPDH (nt 4280–4342) using primers 5′-CGGGGCTCTCCAGAACATC-3′ and 5′-ATGACCTTGCCCACAGCCT-3′ genes were accordingly. Each 10 µL reaction contained 5 ng plasma DNA, 250 nM of each primer, and 5 µL PowerUp SYBR Green Master Mix (Thermo Scientific, Waltham, MA, USA). Cycling conditions were as follows: 50 °C for 2 min, 95 °C for 2 min, followed by 40 cycles of 95 °C for 3 s and 60 °C for 30 s. Product specificity was verified by melt-curve analysis (60–90 °C). All, and all samples were run in triplicate. Standard curves from mtDNA-containing and nDNA-containing vectors of known copy number, no-template controls, and a healthy donor DNA control were included in each run. Absolute copy numbers were derived from respective standard curves and normalized to plasma volume, accounting for extraction and elution volumes and input DNA.

### 4.4. Transendothelial Electrical Resistance Measurements

TEER was measured using a voltohmmeter (EVOM3, World Precision Instruments, Sarasota, FL, USA) with STX4 electrodes. Primary stria vascularis endothelial cells were seeded at 2 × 10^5^ cells/cm^2^ on fibronectin-coated Transwell^®^ membrane inserts with a 0.4 µm pore size (Corning, Corning, NY, USA, cat# 3470). PCs were seeded at 1 × 10^5^ cells/cm^2^ on the opposite (abluminal) side of poly-L-lysine-coated inserts using a two-step seeding protocol. Briefly, after Trypan Blue counting (TC20 automated cell counter, Bio-Rad, Hercules, CA, USA), 150 μL of the PC suspension was applied to the inverted insert (abluminal side) and incubated for 3 h to allow attachment. Inserts were then returned to their upright position in 24-well plates containing PC medium, and 150 μL of endothelial cell suspension was added to the luminal side. After 3 h, wells were filled with endothelial cell medium. Cells were allowed to stabilize for 24 h before the first TEER measurement.

Electrodes were cleaned in 1% Tergazyme^®^ and rinsed with sterile water according to the manufacturer’s instructions and disinfected in 70% ethanol for ≤5 min before use. For each experiment, blank resistance was recorded in inserts filled with medium without cells. TEER was then measured in triplicate in each well once daily by placing the electrode into the apical and basolateral compartments. Net resistance was calculated by subtracting the blank value from the sample resistance, and TEER (Ω·cm^2^) was obtained by multiplying the net resistance by the insert growth area (cm^2^). Electrodes were disinfected with ethanol, rinsed with sterile water, and air-dried after use.

### 4.5. Permeability Assay

Endothelial cells and PCs were cultured on 24-well Transwell^®^ inserts as described above. We added 70 kDa FITC-dextran to the apical compartment after treatment. After 1 h, the medium was collected from the basolateral compartment and fluorescence was measured using a plate reader (excitation 490 nm, emission 520 nm). FITC-dextran concentrations were determined from a standard curve and used to assess paracellular permeability.

### 4.6. LDH Cytotoxicity Assay

Cytotoxicity was assessed using the LDH-Glo™ Cytotoxicity Assay (Promega, Madison, WI, USA, cat# J2381) according to the manufacturer’s instructions. Briefly, 2.5 µL of conditioned medium from treated or control cells was mixed with 47.5 µL LDH Storage Buffer in a white 96-well plate (Corning Costar^®^, Corning, NY, USA, cat# 3917). We then added 50 µL LDH Detection Reagent and incubated the plates for 60 min at room temperature. Luminescence was measured using a Synergy H1 plate reader (BioTek, Winooski, VT, USA).

### 4.7. Fluorescent Staining

For cytoskeletal and syndecan-1 staining, endothelial cells were cultured on human fibronectin-coated 4-well glass-bottom dishes (Ibidi, Gräfelfing, Germany, cat# 80426). Cells were fixed with 4% paraformaldehyde (Sigma, St. Louis, MO, USA, cat# 158127) in phosphate-buffered saline (PBS; Sigma, cat# P4417), permeabilized with 0.1% Triton X-100 (Sigma, cat# X100) in PBS, and incubated for 1 h at room temperature with Alexa Fluor™ 488-conjugated phalloidin (Invitrogen, Carlsbad, CA, USA, cat# A12379) or anti-CD138/syndecan-1 antibody (Invitrogen, cat# MA53-2600). After primary incubation, samples were washed with PBS. For syndecan-1 staining, cells were further incubated with goat anti-rabbit Alexa Fluor™ 488 secondary antibody (Thermo Fisher, Waltham, MA, USA, cat# A-11008) diluted in PBS-T for 1 h at room temperature, followed by washing with PBS. For phalloidin-stained samples, staining was complete after the primary incubation and washing with PBS. In all conditions, nuclei were counterstained with DAPI for 5 min, followed by a final wash with PBS. Coverslips were mounted using Fluorescent Mounting Medium (Dako, Glostrup, Denmark, cat# S3023). Images were acquired using a Nikon Eclipse Ti2 inverted wide-field microscope and processed and analyzed in Fiji (ImageJ, Version: 2.0.0-rc-49/1.51d, Fiji-win32).

### 4.8. Quantification of Plasma Cytokines and Chemokines by ELISA

Circulating cytokine levels were quantified in plasma samples from patients with MD and HCs using commercial ELISA kits according to the manufacturers’ instructions. Human IL-1β and MIP-1α/CCL3 were measured using the Human IL-1β ColorStep ELISA Kit (Assay Genie, Dublin, Ireland, cat# AEFI01732) and Human MIP-1α (CCL3) ColorStep ELISA Kit (Assay Genie, cat# AEFI03335), respectively. Human CCL27/CTACK was quantified using the Human CCL27/CTACK ELISA Kit (Assay Genie, cat# HUFI00059).

Briefly, appropriately diluted plasma samples and serially diluted standards were added in duplicate to pre-coated 96-well plates with the respective capture/detection antibody reagent. After incubation at 37 °C for 60–90 min, depending on the kit, the plates were washed and incubated with HRP-streptavidin or streptavidin-HRP conjugate, followed by TMB substrate to develop the signal. The reaction was stopped with acidic stop solution and absorbance was measured at 450 nm using a microplate reader. Analyte concentrations were calculated from standard curves generated on each plate and expressed as concentration per milliliter of plasma.

### 4.9. Statistical Analysis

Statistical analyses were performed using GraphPad Prism (GraphPad Software version 10.4.1, San Diego, CA, USA). Data normality was assessed with the Shapiro-Wilk test. Differences between the two groups were analyzed using unpaired Student *t*-tests, and multiple-group comparisons were performed by two-way ANOVA. *p*-values < 0.05 were considered significant.

## Data Availability

The raw data supporting the conclusions of this article will be made available by the authors upon request.
